# Towards a neuroscience of social interaction

**DOI:** 10.3389/fnhum.2013.00022

**Published:** 2013-02-01

**Authors:** Ulrich J. Pfeiffer, Bert Timmermans, Kai Vogeley, Chris D. Frith, Leonhard Schilbach

**Affiliations:** ^1^Department of Psychiatry, University Hospital of CologneCologne, Germany; ^2^Wellcome Trust Centre for Neuroimaging, University College LondonLondon, UK; ^3^Max-Planck-Institute for Neurological ResearchCologne, Germany

The burgeoning field of social neuroscience has begun to illuminate the complex biological bases of human social cognitive abilities. However, in spite of being based on the premise of investigating the neural bases of interacting individuals, a majority of studies has focused on studying brains in isolation using paradigms that investigate “offline” social cognition, i.e., social cognition from an observer's point of view, rather than “online” social cognition, i.e., social cognition from an interactor's point of view. Consequently, the neural correlates of real-time social interaction have remained largely elusive and may—paradoxically—be seen to represent the “dark matter” of social neuroscience (Schilbach et al., 2013).

More recently, a growing number of researchers have begun to study social cognition from an interactor's point of view, based on the assumption that there is something fundamentally different when we are actively engaged with others in real-time social interaction as compared to when we merely observe them. Whereas for “offline” social cognition, interaction and feedback are merely a way of gathering data about the other person that feeds into processing algorithms “inside” the agent, it has been proposed that in “online” social interaction the knowledge of the other—at least in part—may reside in the interaction dynamics “between” the agents. Furthermore, being a participant in an interaction may entail a commitment toward being responsive created by important difference in the motivational foundations of “online” and “offline” social cognition.

**Figure 1:**
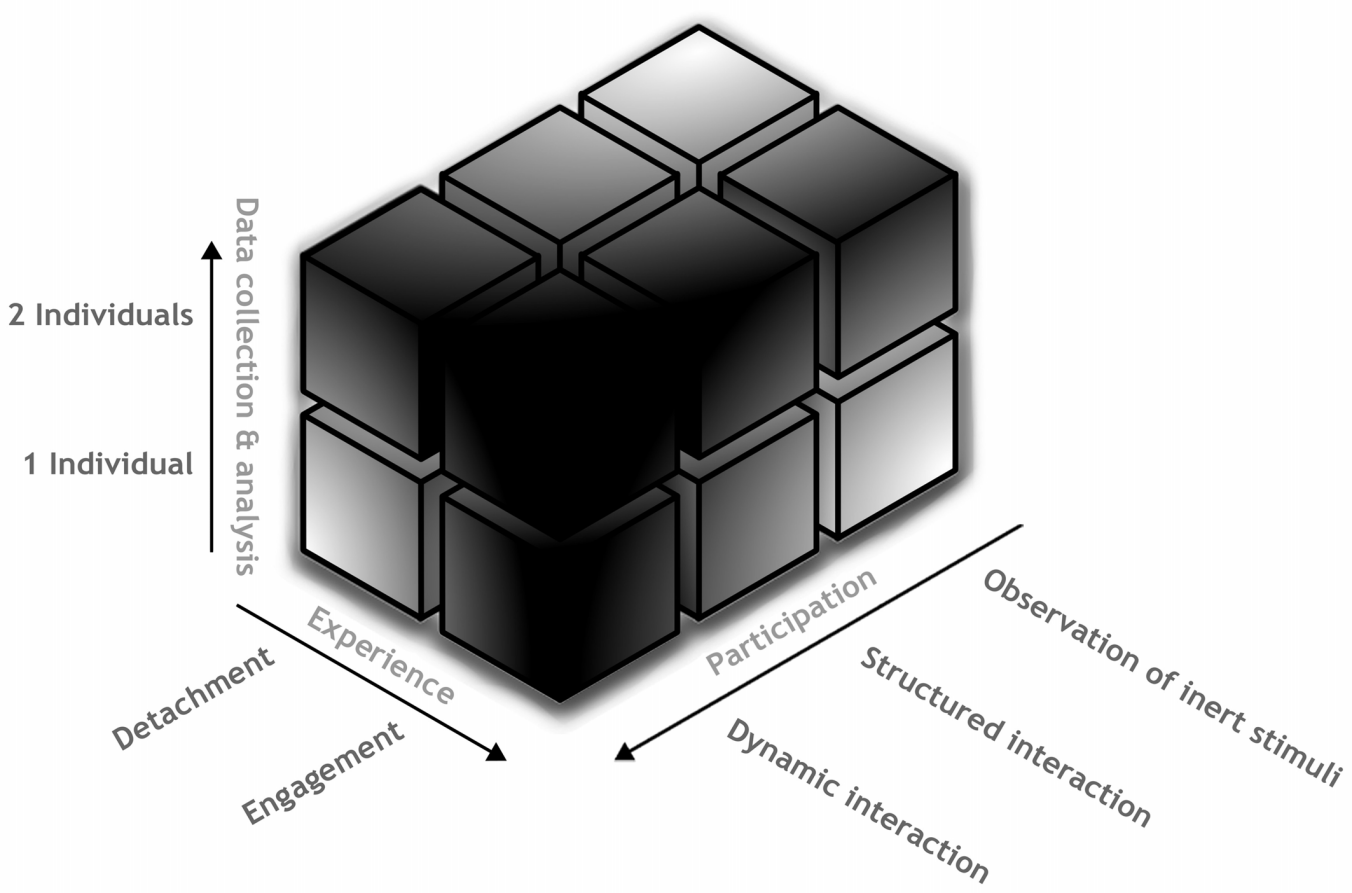
**Depiction of the experimental landscape of research in social neuroscience.** More intense shades of gray indicate areas of the landscape, which have been left largely unexplored, thus, representing the “dark matter” of social neuroscience.

There are at least three different axes along which social neuroscience will have to evolve in order to (a) be able to validate the idea that interaction is more than just an online recruitment of essentially two or more agents' internal social knowledge, and (b) move toward a true understanding of what it is like to exist and function in a social context. In a recent paper (Schilbach et al., 2013; see Figure 1), we describe one axis representing detachment versus emotional engagement; a second axis that runs from purely spectatorial setups to setups that allow participants to produce a meaningful change in their environment, to paradigms in which two agents can interact with each other in a dynamic way; and a third axis that contrasts methodologies that look for explanatory variance within a single agent with approaches focusing on explanatory power of a system of multiple agents. It is important to note that a more enactive approach that incorporates meaningful interaction need not necessarily focus exclusively on dynamic components of ongoing interaction. For instance, establishing the degree to which “passive” social perception and related biobehavioral markers change when in interaction as compared to merely observing, or the study of how we perceive cooperative interaction and adapt to it, is extremely useful and necessary in order to come to a full understanding of social interaction.

In this line of thought, this Frontiers Research Topic brings together contributions from researchers in social neuroscience and related fields, whose work contributes to the development of the neuroscientific investigation of “online” social cognition and draws upon behavioral studies, psychophysiological investigations, computational approaches, developmental, and patient studies while also providing theoretical contributions that can help to advance research in social neuroscience. This creates an interdisciplinary perspective on what it is that separates “online” from “offline” social cognition and how differences in the underlying neurobiological processes and mechanisms can be investigated. The contributions highlight the importance of methodological advances to quantify the interpersonal processes of real-time social interaction and demonstrate how this can be related to measurements obtained from one or two brains.

Without going into each of the 52 contributions to this Research Topic, there are a number of emerging patterns coming to the foreground. All of them, to some degree, focus on at least one aspect of the three axes and try to find an explanation of behavioral variance that cannot be found by exclusively focusing on disengaged agents—be it in engagement, active participation in joint actions, or in the interaction dynamics itself. The theoretical contributions shed light on how recent findings might reveal the crucial and subtle differences between spectatorial versus interactionist social cognition. Moreover, they suggest various ways of conceptualizing this distinction by focusing on coordination dynamics or interactive alignment/synchronization, cooperation, intentionality, brain-computer interfaces, differential involvement of (conscious) top-down processes, and more implicit, automatic processing, or by pointing toward findings in developmental neuroscience.

Among the original research articles, a number focus on neural correlates of some form of live social interaction, either face-to-face, or via gaze and joint attention, joint action in various dual tasks such as imitation, behavioral or listener-speaker coupling. These are not limited to investigating only single agents' neural correlates, but also look at the coupling of participants' neural correlates within an interactive setup. The field of interest pertaining to the nature of interaction stretches far beyond that and incorporates inquiries into risk-taking, inequity, deception—often in the context of games, emotion, and face perception, machine interaction, the role of oxytocin, and specific interaction deficits in persons with autism.

By focusing on cutting-edge research in social neuroscience and related areas, this Frontiers Research Topic allows new insights into the neurobiology of social interaction and demonstrates how the field of social neuroscience is now tackling issues that were at the very heart of the field until its inception, but have proved to be more difficult to assess. Beyond the excellent contributions that make up this Research Topic, we believe that this special focus will also give readers ideas for future research in this field, which—we hope—will continue to turn toward the investigation of phenomena that are inherently linked to participation in social interaction and may therein help social neuroscience to really go social.
